# Prevalence of depressive symptoms and associated factors in Brazilian older adults: 2019 *Brazilian National Health Survey*

**DOI:** 10.1590/0102-311XEN006124

**Published:** 2025-02-07

**Authors:** Frederico Kochen Trevisan, Ritele Hernandez da Silva, Simone Farias Antunez Reis, Marui Weber Corseuil Giehl

**Affiliations:** 1 Curso de Medicina, Universidade Federal de Santa Catarina, Araranguá, Brasil.; 2 Departamento de Ciências da Saúde, Universidade Federal de Santa Catarina, Araranguá, Brasil.; 3 Programa de Pós-graduação em Saúde Coletiva, Universidade Federal de Santa Catarina, Florianópolis, Brasil.

**Keywords:** Depressive Symptoms, Aged, Health Surveys, Sintomas Depressivos, Idoso, Inquéritos Epidemiológicos, Síntomas Depresivos, Anciano, Encuestas Epidemiológicas

## Abstract

This study aimed to describe the prevalence of depressive symptoms and associated factors among older adults. A cross-sectional population-based study using data from the 2019 *Brazilian National Health Survey* was carried out. The prevalence of depressive symptoms was determined using the *9-item Patient Health Questionnaire* (PHQ-9), and associations were tested according to sociodemographic, health and behavioral variables. Crude and adjusted prevalence ratios (PR) with 95% confidence intervals (95%CI) were calculated using Poisson’s regression. The overall prevalence of depressive symptoms was 10.7% (95%CI: 9.9; 11.5). Higher PHQ-9 scores were associated with female gender (PR = 2.11, 95%CI: 1.82; 2.44), lack of participation in religious activities (PR = 1.20, 95%CI: 1.07; 1.35), nonsmoking status (PR = 1.55, 95%CI: 1.32; 1.83), poor or very poor self-perceived health (PR = 7.55, 95%CI: 5.82; 9.80), and multimorbidity (PR = 2.26, 95%CI: 1.85; 2.75). Higher education (PR = 0.55, 95%CI: 0.42; 0.73), income (PR = 0.68, 95%CI: 0.54; 0.85), and physical activity (PR = 0.72, 95%CI: 0.57; 0.90) were found to be negatively associated with the outcome. The most prevalent depressive symptoms were: sleeping problems (24.8%, 95%CI: 23.8; 25.8), not feeling rested or willing/feeling without energy (14.5%, 95%CI: 13.7; 15.4), and being depressed/down/without perspective (10.5%, 95%CI: 9.7; 11.2). These findings highlight the importance of prioritizing the identification and treatment of depressive symptoms in older Brazilian populations, particularly given that one in ten older Brazilians experience depressive symptoms.

## Abstract (pt)

Este estudo buscou descrever a prevalência de sintomas depressivos e seus fatores associados em idosos. Um estudo transversal de base populacional foi realizado com dados da *Pesquisa Nacional de Saúde* de 2019. A prevalência de sintomas depressivos foi determinada pelo *Questionário sobre a Saúde do Paciente de 9 Itens* (PHQ-9) e as associações foram testadas de acordo com variáveis sociodemográficas, de saúde e comportamentais. Razões de prevalência (RP) brutas e ajustadas com intervalos de 95% de confiança (IC95%) foram calculadas pela regressão de Poisson. A prevalência geral de sintomas depressivos foi de 10,7% (IC95%: 9,9; 11,5). Escores mais altos do PHQ-9 foram associados ao sexo feminino (RP = 2,11; IC95%: 1,82; 2,44), falta de participação em atividades religiosas (RP = 1,20; IC95%: 1,07; 1,35), não fumante (RP = 1,55; IC95%: 1,32; 1,83), autopercepção de saúde ruim ou muito ruim (RP = 7,55; IC95%: 5,82; 9,80) e multimorbidade (RP = 2,26; IC95%: 1,85; 2,75). Maiores nível educacional (RP = 0,55; IC95%: 0,42; 0,73), renda (RP = 0,68; IC95%: 0,54; 0,85) e atividade física (RP = 0,72; IC95%: 0,57; 0,90) foram negativamente associados ao desfecho. Os sintomas depressivos mais prevalentes foram problemas de sono (24,8%; IC95%: 23,8; 25,8), não se sentir descansado ou disposto/sentir-se sem energia (14,5%; IC95%: 13,7; 15,4) e estar deprimido/sentindo-se para baixo/sem perspectiva (10,5%; IC95%: 9,7; 11,2). Esses achados destacam a importância de priorizar a identificação e o tratamento de sintomas depressivos em populações idosas brasileiras, principalmente considerando que um em cada dez idosos apresenta sintomas depressivos.

## Abstract (es)

Este estudio buscó describir la prevalencia de síntomas depresivos y sus factores asociados en ancianos. Se realizó un estudio transversal de base poblacional con base en los datos de la *Encuesta Nacional de Salud* de Brasil del 2019. La prevalencia de síntomas depresivos se determinó mediante el *Cuestionario de Salud del Paciente de 9 Ítems* (PHQ-9) y se probaron las asociaciones según variables sociodemográficas, de salud y de conducta. Las razones de prevalencias (RP) brutas y ajustadas con intervalos de 95% de confianza (IC95%) se calcularon mediante la regresión de Poisson. La prevalencia general de síntomas depresivos fue del 10,7% (IC95%: 9,9; 11,5). Las puntuaciones más altas del PHQ-9 se asociaron con el sexo femenino (RP = 2,11; IC95%: 1,82; 2,44), falta de participación en actividades religiosas (RP = 1,20; IC95%: 1,07; 1,35), no fumador (RP = 1,55; IC95%: 1,32; 1,83), autopercepción de salud mala o muy mala (RP = 7,55; IC95%: 5,82; 9,80) y multimorbilidad (RP = 2,26; IC95%: 1,85; 2,75). Mayores niveles educativos (RP = 0,55; IC95%: 0,42; 0,73), ingresos (RP = 0,68; IC95%: 0,54; 0,85) y actividad física (RP = 0,72; IC95%: 0,57; 0,90) se asociaron negativamente con el desenlace. Los síntomas depresivos más prevalentes fueron problemas para dormir (24,8%; IC95%: 23,8; 25,8), no sentirse descansado o dispuesto/sentirse sin energía (14,5%; IC95%: 13,7; 15,4) y estar deprimido/sentirse pesimista/sin perspectiva (10,5%; IC95%: 9,7; 11,2). Estos hallazgos resaltan la importancia de priorizar la identificación y el tratamiento de los síntomas depresivos en la población brasileña de ancianos, especialmente considerando que uno de cada diez ancianos presenta síntomas depresivos.

## Introduction

Brazil is currently experiencing a demographic transition, characterized by a decline in both fertility and mortality rates. This demographic transition has resulted in an inversion of the age pyramid, with an increased proportion of older individuals relative to the total population. The latest Brazilian census data indicates that by 2060, the number individuals aged 60 and above will reach 58.4 million, corresponding to 26.7% of the total population ^1^.

In light of this observation, it can be reasonably inferred that the consequences of aging will have an even greater impact on the Brazilian population in the coming years, as chronic diseases become more prevalent in this age group. With advancing age, a range of physical and mental challenges may arise, including chronic conditions, psychological disorders, and dementia ^2^.

Mental disorders such as depression are a major contributor to these problems. As a result, the number of older adults experiencing depressive symptoms is expected to increase ^3^ ^,^ ^4^. Depression, one of the most common and important physiological and psychological changes among psychiatric disorders, is frequently diagnosed in older people and negatively affects their quality of life, function, and autonomy. These challenges often result in frequent hospitalizations, reduced life expectancy, physical illness and loss of independence ^5^ ^,^ ^6^.

A systematic review indicates that the global prevalence of depression in older adults was 28.4%, ranging from 8.2% to 53.1%. These figures show a considerable degree of heterogeneity between studies ^4^. In addition, these discrepancies can be attributed to differences in study areas, perceptions of mental health problems, availability and quality of basic public health services, and use of screening tools ^4^. Among Brazilian older people, the global prevalence of depressive symptoms was 21%, with significant regional variations, ranging from 7% to 39% ^7^.

Prior research has demonstrated that individuals of advanced age, female gender, lower levels of schooling and income are more likely to experience depressive symptoms among older adults. Moreover, low levels of physical activity and social support, in addition to habits such as smoking and alcohol consumption, have been identified as contributing factors to the mental health of this population ^2^ ^,^ ^8^ ^,^ ^9^ ^,^ ^10^ ^,^ ^11^. Regarding health conditions, the presence of chronic diseases, including cardiovascular issues, diabetes, and respiratory diseases, as well as, a poorer self-perception of health, are consistently linked to an increased risk of depressive symptoms in this age group ^2^ ^,^ ^12^ ^,^ ^13^.

Given evidence indicating that the prevalence of depressive symptoms increases morbidity, lowers quality of life, and results in higher suicide and all-cause mortality rates (e.g., by increasing the risk of cardiovascular mortality) ^14^, this issue represents an emerging public health concern. A comprehensive understanding of the factors associated with depression in older adults is essential for the identification of high-risk groups and the mitigation of risk factors. This knowledge can inform the development of effective preventive interventions, early treatment protocols and follow-up strategies aimed at reducing the prevalence and severity of depressive symptoms. Furthermore, a deeper comprehension of these factors may contribute to the promotion of mental health and healthy aging in the older population.

Therefore, this paper aims to analyze the prevalence of depressive symptoms and the factors associated with this outcome in older Brazilians, based on data from the *Brazilian National Health Survey* (PNS, acronym in Portuguese) conducted in 2019.

## Method

This study used a cross-sectional population data from the PNS 2019 ^15^, which included a representative sample of the Brazilian population living in private households throughout the country. The PNS is carried out by the Brazilian Ministry of Health, in partnership with the Brazilian Institute of Geography and Statistics (IBGE, acronym in Portuguese). The sampling process was carried out in three stages: (i) first, census tracts were drawn, (ii) then households, and finally, (iii) one adult resident aged 15 or over was randomly selected among all the adult residents of the household. The weighting factors were calculated for each of the three sampling units, taking into account the probabilities of selection and the estimated nonresponse rate. The total number of households sampled was 108,525, and interviews were carried out in 94,114 households. The PNS questionnaires include three sections: (i) a questionnaire relating to the characteristics of the household, (ii) another relating to the residents, and (iii) a third specific to the selected resident. Further details on the PNS 2019 sampling process and methods have been previously published ^15^.

This study analyzed data from 22,728 older adults aged 60 or over, who answered the questionnaire for the selected resident.

### Outcome variable: depressive symptoms

The presence of depressive symptoms was evaluated using the *Patient Health Questionnaire-9* (PHQ-9), which assesses the frequency of depressive symptoms experienced within the two weeks prior to the interview. The instrument has been validated for use in the Brazilian population ^16^, with applicability in the diagnosis of depression at cut-off points > 9 and > 10. The depressive symptoms were classified in accordance with the recommendations set forth by Kroenke et al. ^17^, which employ a severity classification system based on the following thresholds: none (1-4 points), mild (5-9), moderate (10-14), moderately severe (15-19), and severe (20-27 points). In this study, the presence of depressive symptoms was defined by a score of 10 or more on the PHQ-9, which has been identified as the optimal cut-off point for detecting clinically relevant symptoms ^18^ ^,^ ^19^.

### Exposure variables

The selection of the exposure variables was based on the previously published literature ^4^ ^,^ ^7^ ^,^ ^11^ ^,^ ^12^ ^,^ ^13^. The aim was to identify the complexity of factors associated with the presence of depressive symptoms among older adults. The most frequently associated factors were sociodemographic characteristics (age, gender), poor socioeconomic conditions, unhealthy lifestyle, and comorbidities, among others.

The sociodemographic variables assessed included gender (male; female), age group in years (60-69; 70-79; ≥ 80), race/skin color (white; black/mixed), marital status (married; divorced/single; widowed); schooling (illiterate or incomplete elementary school; complete elementary school or incomplete high school; complete high school or incomplete higher education; complete higher education), per capita income of the family, classified in minimum wages (≤ 1; 1-3; > 3).

The variables classified as social support were analyzed, including the frequency of engaging in physical activity with other individuals, participation in social and/or community groups, involvement in volunteer work, and religious activities. These variables were dichotomized (i.e., classified as either frequent or infrequent).

The health-related behaviors included in the study were sufficient physical activity during leisure time, current smoking status (yes or no), and alcohol consumption (never, less than once a month, or once a month or more). Individuals who reached 150 minutes of light or moderate intensity exercise a week, or 75 minutes of vigorous intensity exercise a week, regardless of the number of days they did it a week, were considered physically active ^20^.

Additionally, health conditions were incorporated into the analysis, including self-perceived health status (classified as very good/good, regular, or poor/very poor), and the presence of multimorbidity, which was evaluated using a cutoff of ≥ 2 diseases from a list of morbidities available in the PNS. These were investigated through the use of self-reported medical diagnoses. To ascertain the presence of each disease based on self-reported medical diagnoses, the following question was employed: “Has a physician ever diagnosed you as having (each disease)?”. The following diseases were included in this study: hypertension, diabetes, high cholesterol, heart disease, stroke, asthma, arthritis or rheumatism, chronic back problems, depression, mental illness (schizophrenia, bipolar disorder or compulsive-obsessive disorder), lung disease (chronic bronchitis, emphysema or chronic obstructive pulmonary disease), cancer, chronic renal failure and work-related musculoskeletal disorders.

The data were processed and analyzed using the Stata 14.2 software (https://www.stata.com). The design effect resulting from the complex sampling process was accounted for in all analyses with the prefix “*svy*” employed. The sample was described using both absolute and relative frequencies. Additionally, prevalence rates and corresponding 95% confidence intervals (95%CI) were calculated for the outcome based on the exposure variables (chi-square test). Both unadjusted and adjusted analyses were conducted using Poisson’s regression, with prevalence ratios (PR) estimated at a 5% significance level. In the adjusted analysis, the variables were included by level, considering a hierarchical model of determination ^21^. The first level included gender, age, skin color, and marital status. The second level included variables related to education and per capita income. The third level comprised variables pertaining to social support variables, health-related behaviors, and health conditions. After adjusting for the other variables within the same block and those hierarchically higher, associations with a p-value < 0.05 were considered statistically significant. To control for potential confounding factors, variables with p < 0.20 were retained in the final model. All the analyses accounted for the effect of the study design and sample weights.

The PNS was approved by the Brazilian National Research Ethics Committee (process n. 3.529.376, dated August 23, 2019). All interviewees signed an informed consent form. The PNS 2019 database and questionnaire modules are available for public access and use on the survey website ^22^.

## Results

Data from 22,728 individuals were analyzed, for 70.1 years (± 8.9) as the mean age, the majority of whom were female (56.7%), more than half were aged between 60 and 69 years (56.3%). Most individuals self-identified as white (51.4%), and were married (50.7%). Furthermore, 63.3% of the older adults had incomplete or no primary education, approximately one-tenth had completed higher education (11.3%), and 42.7% had an income of up to three minimum wages. Regarding social support, 77.6% of the older adults reported infrequent support for physical activity, and almost of them (95.3% and 94.4%, respectively) reported little participation in community groups or voluntary work. However, regarding social support in religious activities, almost half of them (48.5%) took part in collective activities related to the religion they practiced (Table 1).

**Table 1:** Sociodemographic and economic characteristics according to the prevalence of depressive symptoms in Brazilian older adults. *Brazilian National Health Survey*, 2019.

Variables	n (%)	Prevalence of depressive symptoms
% (95CI%)
Gender		
Male	10,193 (43.3)	6.6 (5.7; 7.5) *
Female	12,535 (56.7)	13.9 (12.9; 15.0) *
Age (years)		
60-69	12,555 (56.3)	10.6 (9.3; 12.0) **
70-79	7,157 (30.1)	10.3 (8.9; 11.8) **
≥ 80	3,016 (13.6)	11.0 (10.0; 12.2) **
Race/Skin color		
White	9,901 (51.4)	10.8 (9.7; 12.0)
Mixed/Black	12,456 (48.6)	10.6 (9.8; 11.6)
Marital status		
Married	9,946 (50.7)	9.1 (8.1; 10.1) *
Divorced/Single	6,698 (24.3)	11.6 (10.2; 13.3) *
Widowed	6,084 (25.0)	13.2 (11.8; 14.7) *
Schooling		
Illiterate or incomplete elementary school	14,987 (63.3)	12.3 (11.4; 13.3) *
Complete elementary or incomplete high school	2,011 (9.5)	9.8 (7.8; 12.2) *
Complete high school or incomplete higher education	3,322 (15.9)	8.7 (7.1; 10.5)
Complete higher education	2,408 (11.3)	5.4 (4.2; 6.8) *
Per capita income (minimum wages)		
≤ 1	10,250 (41.7)	12.7 (11.5; 13.9) *
1-3	8,904 (42.7)	10.5 (9.3; 11.7) *
> 3	3,571 (15.6)	6.3 (5.2; 7.6) *
Social support for physical activity		
Frequent	4,921 (22.4)	6.5 (5.5; 7.7) *
Infrequent	17,807 (77.6)	11.9 (11.1; 12.9) *
Support social groups		
Frequent	1,059 (4.7)	8.3 (5.7; 11.8)
Infrequent	21,669 (95.3)	10.8 (10.1; 11.6)
Support volunteer work		
Frequent	1,158 (5.6)	8.9 (6.4; 12.1)
Infrequent	21,570 (94.4)	10.8 (10.1; 11.7)
Support religious activities		
Frequent	10,892 (48.5)	9.31 (8.4; 10.3) *
Infrequent	11,836 (51.5)	12.0 (11.0; 13.2) *
Leisure-time physical activity		
Insufficiently active	18,517 (80.5)	12.1 (11.3; 13.1) *
Active	4,211 (19.5)	4.8 (4.0; 5.9) *
Current smoker		
Yes	2,680 (11.4)	16.2 (13.7; 19.1) *
No	20,048 (88.6)	10.0 (9.3; 10.8) *
Alcohol consumption		
Never	17,096 (73.3)	12.3 (11.4; 13.2) **
< Once a month	1,877 (7.8)	6.6 (5.0; 8.5) **
> Once a month	3,755 (18.9)	6.4 (5.1; 7.9) **
Self-perceived health		
Very good/Good	10,210 (47.1)	3.3 (2.7; 4.0) *
Regular	9,777 (41.7)	11.6 (10.5; 12.9) *
Poor/Very poor	2,741 (11.2)	38.4 (35.3; 41.7) *
Multimorbidity		
< 2 morbidities	9,682 (41.9)	4.2 (3.6; 5.0) *
≥ 2 morbidities	12,043 (58.1)	15.6 (14.4; 16.9) *

95CI%: 95% confidence interval.

* p < 0.001;

** p < 0.05.

Regarding lifestyle habits, 80.5% of those interviewed were classified as insufficiently active, approximately 90% reported not being smokers and more than 80% had normal alcohol consumption. In terms of health characteristics, nearly half of the older adults rated their health as good or very good, whereas 58.1% were classified as having multimorbidity (two or more chronic diseases) (Table 1).

The overall prevalence of depressive symptoms in the sample of older adults investigated was 10.7% (95%CI: 9.9; 11.5). Table 1 illustrates the prevalence of depressive symptoms according to the categories of exposure variables. It demonstrates that the outcome is more prevalent among women, older individuals, those who reported being widowed, and those with lower levels of schooling and income. In addition, the prevalence of depressive symptoms was higher among individuals with limited support for physical activity and religious activities, as well as in those who were physically inactive, smokers, those who perceived their health as poor or very poor, and those with multimorbidity.

Table 2 shows the prevalence of the most commonly reported depressive symptoms, considering two weeks as a parameter and their presence on more than half of the days. Approximately 25% (95%CI: 23.8; 25.8) of the older adults experienced sleep problems, 14.5% (95%CI: 13.7; 15.4) reported problems with not feeling rested, and 9.6% (95%CI: 8.8; 10.3) found lower interest or pleasure in engaging activities. Additionally, 8.8% (95%CI: 8.2; 9.5) of the older adults experienced eating problems, 8% (95%CI: 7.4; 8.7) experienced slowness of movement, speech, or agitation, and 10.5% (95%CI: 9.7; 11.2) reported feelings of depression, sadness or a lack of perspective.

**Table 2:** Characteristics of self-reported depressive symptoms in Brazilian older adults. *Brazilian National Health Survey*, 2019.

Variables	% (95%CI)
Sleep problem	
< half the days	75.2 (74.2; 76.2)
> half the days	24.8 (23.8; 25.8)
Problems with not feeling rested or in the mood/no energy	
< half the days	85.5 (84.6; 86.3)
> half the days	14.5 (13.7; 15.4)
Little interest/pleasure in doing things	
< half the days	90.4 (89.7; 91.1)
> half the days	9.6 (8.8; 10.3)
Trouble concentrating on usual activities	
< half the days	92.5 (91.8; 93.1)
> half the days	7.52 (6.9; 8.2)
Eating problems (lack or excess of appetite)	
< half the days	91.2 (90.5; 91.8)
> half the days	8.8 (8.2; 9.5)
Slowness in moving or speaking or agitation	
< half the days	92.0 (91.3;92.6)
> half the days	8.0 (7.4; 8.7)
Depressed/Down/No perspective	
< half the days	89.5 (88.8; 90.3)
> half the days	10.5 (9.7; 11.2)
Feeling bad about yourself/thinking you’re a failure	
< half the days	95.2 (94.7; 95.7)
> half the days	4.8 (4.3; 5.3)
Thought about hurting yourself/it would be better to be dead	
< half the days	98.4 (98.1; 98.6)
> half the days	1.6 (1.4; 1.9)

95%CI: 95% confidence interval.

Table 3 presents the unadjusted and adjusted analyses for the association between the exposure variables and the outcome. In the unadjusted analysis, all the sociodemographic variables, lifestyle factors, and health conditions were found to be associated with the prevalence of depressive symptoms. With regard to social support, only support for physical activity and participation in religious activities were associated with the outcome. The adjusted analysis revealed that women exhibited a prevalence of depressive symptoms approximately two times higher than men (PR = 2.11, 95%CI: 1.83; 2.45). Older adults with higher levels of education (PR = 0.55, 95%CI: 0.42; 0.73), greater income (PR = 0.68, 95%CI: 0.54; 0.85), and engagement in leisure-time physical activity (PR = 0.72, 95%CI: 0.57; 0.90) exhibited a lower prevalence of depressive symptoms. A higher prevalence of depressive symptoms was observed among older adults who were unengaged in religious activities activity (PR = 1.20, 95%CI: 1.07; 1.35) and nonsmokers (PR = 1.55, 95%CI: 1.32; 1.83). Furthermore, older individuals with a poor or very poor perceived state of health had a higher prevalence of depressive symptoms (PR = 7.55, 95%CI: 5.82; 9.80), as did those with multimorbidity (PR = 2.26, 95%CI: 1.85; 2.75).

**Table 3:** Crude and adjusted analysis of the association between depressive symptoms and demographic, socioeconomic, behavioral, and health variables in Brazilian older adults. *Brazilian National Health Survey*, 2019.

	Crude		Adjusted	
	PR (95%CI)	p-value	PR (95%CI)	p-value
Level 1				
Gender		< 0.001		< 0.001
Male	1.00		1.00	
Female	2.12 (1.83; 2.45)		2.11 (1.82; 2.44)	
Age (years)		0.061		0.110
60-69	1.00		1.00	
70-79	0.97 (0.84; 1.11)		0.96 (0.83; 1.11)	
≥ 80	1.26 (1.05; 1.51)		1.22 (1.02; 1.46)	
Race/Skin color		0.786		
White	1.00			
Mixed/Black	0.98 (0.86; 1.12)			
Marital status		< 0.001		
Married	1.00			
Divorced/Single	1.28 (1.08; 1.53)			
Widowed	1.46 (1.26; 1.69)			
Level 2				
Schooling		< 0.001		< 0.001
Illiterate or incomplete elementary school	1.00		1.00	
Complete elementary or incomplete high school	0.79 (0.63; 0.99)		0.84 (0.66; 1.05)	
Complete high school or incomplete higher education	0.70 (0.58; 0.86)		0.79 (0.64; 0.97)	
Complete higher education	0.44 (0.34; 0.56)		0.55 (0.42; 0.73)	
Per capita income (minmum wages)		< 0.001		0.002
≤ 1	1.00		1.00	
1-3	0.83 (0.72; 0.95)		0.88 (0.76; 1.02)	
> 3	0.50 (0.40; 0.61)		0.68 (0.54; 0.85)	
Level 3				
Social support for physical activity		< 0.001		0.135
Frequent	1.00		1.00	
Infrequent	1.83 (1.53; 2.18)		1.15 (0.96; 1.37)	
Support social groups		0.141		
Frequent	1.00			
Infrequent	1.31 (0.91; 1.87)			
Support volunteer work		0.228		
Frequent	1.00			
Infrequent	1.22 (0.88; 1.68)			
Support religious activities		< 0.001		0.003
Frequent	1.00		1.00	
Infrequent	1.29 (1.13; 1.47)		1.20 (1.07; 1.35)	
Leisure-time physical activity		< 0.001		0.004
Insufficiently active	1.00		1.00	
Active	0.40 (0.32; 0.49)		0.72 (0.57; 0.90)	
Current smoker		< 0.001		< 0.001
Yes	1.00		1.00	
No	1.62 (1.35; 1.93)		1.55 (1.32; 1.83)	
Alcohol consumption		< 0.001		
Never	1.00			
< Once a month	0.54 (0.41; 0.70)			
> Once a month	0.12 (0.11; 0.13)			
Self-perceived health		< 0.001		< 0.001
Very good/Good	1.00		1.00	
Regular	3.53 (2.86; 4.35)		2.76 (2.19; 3.48)	
Poor/Very poor	11.66 (9.40; 14.46)		7.55 (5.82; 9.80)	
Multimorbidity		< 0.001		< 0.001
< 2 morbidities	1.00		1.00	
≥ 2 morbidities	3.68 (3.04; 4.44)		2.26 (1.85; 2.75)	

95CI%: 95% confidence interval; PR: prevalence ratio.

## Discussion

The prevalence of depressive symptoms was observed in approximately 10% of the studied population. The population groups with the highest frequency of symptoms were women, individuals with low levels of education and income, and those without any type of frequent social support. Regarding health behaviors, the study revealed positive associations between the outcome and physical inactivity, poorer self-perceived health, multimorbidity, and non-smoking status.

An association between the female gender and the presence of depressive symptoms has been previously documented in the literature ^3^ ^,^ ^6^ ^,^ ^23^ ^,^ ^24^ ^,^ ^25^. Note that potentially stressful events may exacerbate the vulnerability to mental health problems in older women, which can be precipitated by gender inequality and the social roles they occupy within the family ^3^ ^,^ ^24^. For example, research indicates that women have less access to education and later mortality compared to men, which results in fewer job opportunities, lower income, and increased exposure to bereavement ^26^. Additionally, hormones such as estrogen play a significant role in mood regulation, suggesting that hormonal deficiencies in older women may contribute to their increased vulnerability to depression ^3^ ^,^ ^27^.

With regard to socioeconomic factors, a consistent association has been observed between higher schooling levels and lower prevalence of depressive symptoms ^6^ ^,^ ^23^. The acquisition of knowledge through schooling can influence cognitive development, coping skills, and access to socioeconomic resources, all of which are related to mental health. The literature indicates that a higher schooling level plays an important protective role against depression in older adults ^6^ ^,^ ^28^. Higher educational attainment in older adults can influence the development of cognitive skills, access to social and economic resources, greater autonomy, and healthy behaviors, thereby positively affecting their mental health ^29^ ^,^ ^30^.

Socioeconomic disadvantage is a well-established risk factor for depression ^6^ ^,^ ^31^. There is evidence of a bidirectional relationship between economic losses and depressive symptoms ^6^. Similarly, social position, which is shaped by the economic situation, is inversely related to some depressive symptoms in both Brazilian and international studies ^32^ ^,^ ^33^ ^,^ ^34^ ^,^ ^35^. This results in feelings of sadness when thinking about the past and exclusion from social life to save money ^34^ ^,^ ^35^.

Another significant correlation of depressive symptoms was involvement in religious groups, which served as a source of social support and engagement within the community. A systematic review that sought to elucidate the association between social support and depression among Asian older adults revealed that individuals who reported higher levels of general social support, such as having a spouse or partner, having a large social network, and access to either emotional or instrumental support, exhibited a reduced prevalence of depressive symptoms compared to those who did not receive the same level of support ^36^. The relationship between depressive symptoms and social support among older adults highlights the importance of therapeutic and preventive strategies that integrate social support as a fundamental aspect of mental health care for this population. Interventions centered on enhancing social support, such as support groups and community activities, have demonstrated efficacy in mitigating depressive symptoms and improving the psychological well-being among older adults.

Regarding smoking, a discrepancy between the findings of different studies is evident, particularly with respect to the methodology employed. Some studies have indicated a positive association between smoking and depressive symptoms ^37^ ^,^ ^38^. Numerous hypotheses suggest a relationship between smoking and depressive symptoms. It is proposed that smoking acts as a self-medication for feelings of sadness and negative mood, consequently leading to a need to smoke to relieve these symptoms. There is evidence that nicotine, a constituent of in tobacco, has an impact on the neuro-regulatory system, which supports the hypothesis that mood changes take place ^38^. However, few studies have reached the same conclusion ^2^, with others not indicating the same association ^6^.

Additionally, older adults with the poorest self-perceived health status tend to exhibit the most pronounced symptoms ^6^ ^,^ ^24^ ^,^ ^39^. The literature provides evidence that the observed association is a consequence of the perception of functional losses associated with the aging process ^3^. The decline in cognitive function, the absence or reduction of a sexually active and working life, and the experience of uselessness brought on by functional incapacity, collectively exacerbate the individual’s perception of health ^3^ ^,^ ^24^ ^,^ ^40^ ^,^ ^41^. Although self-perceived health is a subjective indicator, the literature notes that it is influenced by various conditions, including family support, marital status, educational opportunities, income, functional capacity, chronic health conditions, and lifestyle ^28^ ^,^ ^42^.

This study found that older people with multimorbidity, defined as the coexistence of two or more chronic diseases, exhibit a higher prevalence of depressive symptoms. This result aligns with previous research studies ^2^ ^,^ ^5^ ^,^ ^43^ ^,^ ^44^. A review study identified a bidirectional relationship between multimorbidity and depressive symptoms, indicating that the presence of multiple chronic diseases can increase the risk of developing depressive symptoms, whereas depression can exacerbate the course of chronic diseases. These effects may be attributed to brain function alterations or psychological and psychosocial changes ^28^ ^,^ ^45^. This creates a vicious circle between depressive feelings, physical comorbidities ^37^, worsening quality of life, and prognosis. This highlights the need to assess and treat psychological aspects in patients with multiple clinical conditions ^46^.

In examining the characteristics of self-reported depressive symptoms among Brazilians older adults, this study identified a high prevalence of sleep-related issues, including difficulties with initiating and maintaining sleep, feelings or restlessness or fatigue, and low energy levels. Regarding sleep disorders, the literature indicates that insomnia is the primary characteristic of depressive disorders. It may precede the onset of depressive symptoms, manifest in the early stages of depressive illness ^47^. Some studies indicate that sleep disorders may be present in almost 80% of cases of depression, affecting both the quality and quantity of sleep ^48^. The main complaints include difficulty initiating sleep, reduction in total sleep time, nocturnal and early awakenings in the morning, and disturbing dreams ^47^.

Fatigue is a common phenomenon experienced by all humans. In psychological literature, it is defined as a state of weariness accompanied by a reduction in motivation ^49^. Moreover, it is a symptom found in over 90% of patients with major depressive disorder ^49^. The literature provides evidence that fatigue is one of the five objective criteria associated with the aging process, collectively known as a geriatric frailty syndrome ^50^. Consequently, frailty is used to describe a clinical condition that is unfavorable to the older adults, emphasizing the association between aging and depressive symptoms ^51^.

In this study, it was found that a depressed mood, which is characterized by intense sadness, melancholy, feelings of low mood or lack of hope, was one of the most commonly reported symptoms in individuals with any depressive symptom. This is because the term “depression” is currently defined in such a way that it encompasses both a normal affective state (i.e., a depressed state) and a sign, symptom, syndrome, or illness ^52^. Another factor that contributes to the confusion surrounding the term is that, according to the DSM-5 (*Diagnostic and Statistical Manual of Mental Disorders*, 5th edition) criteria, a depressed mood is one of the two mandatory criteria for a diagnosis of depression ^53^.

This study’s primary strengths are its population-based sample and its representation of Brazilian older adults, a population that has been under-researched in the Brazilian context. Furthermore, the items used to assess depressive symptoms are consistent with those employed in other studies on this topic. However, it is important to consider the limitations of the study when interpreting the results. Primarily, the cross-sectional design does not allow for the identification of a causal link between the exploratory variables and the investigated outcome. Nevertheless, it does indicate the magnitude of the associations, and could bring new approaches to the development of the study area. Furthermore, the use of self-reported measures of depressive symptoms and other health conditions may result in an overestimation of the prevalence of these variables.

## Conclusion

The aim of this study was to examine the prevalence of depressive symptoms and their associated factors among older adults, based on the PNS 2019 data set. This enabled the identification of the prevalence of sleep problems (including both insomnia and hypersomnia), mood disorders, and depressed mood, as measured by the PHQ-9, among the most common depressive symptoms. The findings indicated that female gender, lack of social support for physical activity, no-smoking status, poorer self-perceived health, and multimorbidity were positively associated with a higher prevalence of depressive symptoms among older adults. In contrast, socioeconomic factors were inversely associated with the outcome, reducing its occurrence. Finally, given that depression is one of the most prevalent mental conditions among older adults and has a significant impact on their quality of life, function and autonomy, meaning it is essential to prioritize the identification of these depressive symptoms and their associated factors among the older individuals in order to inform policy development.
